# Data-Driven Multidimensional Clinical Phenotypes and Longitudinal Changes in Type 2 Diabetes Mellitus: A Retrospective Cohort Study

**DOI:** 10.3390/biomedicines14091903

**Published:** 2026-08-26

**Authors:** Andreea Diana Igna, Bianca-Lăcrimioara Petca, Paula-Alexandra Popovici, Timea Claudia Ghitea, Mihaela Simona Popoviciu

**Affiliations:** 1 Doctoral School of Biological and Biomedical Sciences, University of Oradea, 1 University Street, 410087 Oradea, Romania; igna.andreeadiana@student.uoradea.ro (A.D.I.); criste.biancalacrimioara@student.uoradea.ro (B.-L.P.); cipleu.paulaalexandra@student.uoradea.ro (P.-A.P.); 2 Pharmacy Department, Faculty of Medicine and Pharmacy, University of Oradea, 1 University Street, 410087 Oradea, Romania; 3 Department of Preclinical Disciplines, Faculty of Medicine and Pharmacy, University of Oradea, 410028 Oradea, Romania; mihaela.popoviciu@didactic.uoradea.ro

**Keywords:** type 2 diabetes mellitus, clinical phenotyping, cluster analysis, precision medicine, cardiorenal disease, inflammation, TyG index, FIB-4, urinary albumin-to-creatinine ratio, chronic kidney disease, personalized medicine

## Abstract

**Background:** Type 2 diabetes mellitus (T2DM) is clinically heterogeneous, and routinely available renal, inflammatory, and hepatic measures may add information beyond glycemic assessment. **Methods:** Among 811 screened adults, 809 had a standardized baseline assessment and six-month follow-up; two were excluded because a required baseline clustering variable was missing, leaving 807 complete cases for phenotype derivation. Twelve-month data were available for 320 participants. Nine baseline variables were standardized, with UACR and CRP analyzed after ln(x + 1) transformation. Candidate two- to six-cluster solutions were assessed using silhouette, Calinski–Harabasz, and Davies–Bouldin indices, 200 bootstrap resamples, and a consistent-transformation sensitivity analysis. Longitudinal outcomes were evaluated using generalized estimating equations including time, phenotype, and their interaction. **Results:** The final stored four-cluster solution comprised a comparatively favorable-profile phenotype (*n* = 294), a cardiorenal–fibrotic phenotype (*n* = 239), an inflammatory–metabolic phenotype (*n* = 141), and a severe hyperglycemic–obesity phenotype (*n* = 133). Internal separation was modest (silhouette 0.118; Calinski–Harabasz 87.6; Davies–Bouldin 2.118), bootstrap agreement was moderate (median adjusted Rand index 0.445), and the consistent ln(x + 1) sensitivity solution showed substantial agreement with the stored assignment (adjusted Rand index 0.776). Significant phenotype-by-time interactions were observed for HbA1c, BMI, TyG, triglycerides, UACR, eGFR, FIB-4, and CRP after false-discovery-rate correction, but not for LDL cholesterol. Baseline characteristics did not differ detectably between participants with and without twelve-month follow-up after correction. Marked treatment changes occurred by T2, particularly increased use of GLP-1 receptor agonists and SGLT2 inhibitors. **Conclusions:** The four phenotypes provide an exploratory multidimensional description of this cohort. Longitudinal differences should not be attributed to phenotype biology alone because regression to the mean and treatment intensification are plausible contributors; external validation remains necessary.

## 1. Introduction

Type 2 diabetes mellitus (T2DM) is a highly heterogeneous metabolic disorder characterized by substantial interindividual variability in clinical presentation, disease progression, therapeutic response, and risk of chronic complications. Although glycated hemoglobin (HbA1c) remains the cornerstone of diabetes monitoring, patients with similar HbA1c values frequently exhibit markedly different cardiometabolic, renal, inflammatory, and hepatic profiles, highlighting the limitations of glycemia-centered risk assessment alone [1,2].

Current international guidelines advocate individualized therapeutic strategies based not only on glycemic control but also on the presence of cardiovascular disease, chronic kidney disease, obesity, and other comorbidities. Consequently, the therapeutic landscape of T2DM has shifted from a glucose-centric approach toward integrated cardiorenal-metabolic management. However, in routine clinical practice, treatment decisions are often influenced by multiple interacting patient characteristics, making it challenging to identify the dominant clinical phenotype that should guide personalized therapy [3,4].

The seminal data-driven classification by Ahlqvist et al. identified five adult-onset diabetes subgroups—severe autoimmune diabetes, severe insulin-deficient diabetes, severe insulin-resistant diabetes, mild obesity-related diabetes, and mild age-related diabetes—using age at diagnosis, BMI, HbA1c, glutamic acid decarboxylase antibodies, and HOMA2 estimates of beta-cell function and insulin resistance [5]. This framework has been replicated in other populations, including the Danish DD2 cohort [6]. Many, although not all, subsequent phenotyping studies have continued to emphasize glycemic, anthropometric, insulin-secretion, or insulin-resistance variables; fewer routine-care models combine renal function, albuminuria, systemic inflammation, and hepatic fibrosis in one clustering framework [7,8,9]. The present approach is therefore intended to complement, not replace, the established Ahlqvist framework.

Routine clinical datasets provide an opportunity to construct pragmatic phenotyping models that reflect everyday diabetes care. Variables such as UACR, eGFR, the TyG index, BMI, CRP, and FIB-4 are increasingly available in outpatient records and may extend descriptions based mainly on glycemia, adiposity, and inferred insulin resistance [10,11,12]. This added breadth may help identify dominant measured burdens, but it does not establish biological subtypes or future risk categories.

Rather than considering individual biomarkers in isolation, a multidimensional phenotyping strategy may capture the complex biological interactions underlying T2DM and provide a framework for more personalized risk stratification. Such an approach may also help explain why patients with apparently similar glycemic control often exhibit markedly different patterns of renal function, metabolic deterioration, or cardiovascular burden.

The present study aimed to identify multidimensional clinical phenotypes using nine routinely available baseline variables representing diabetes chronicity, glycemic severity, adiposity, insulin resistance, lipid status, renal function, albuminuria, hepatic fibrosis, and systemic inflammation.

## 2. Materials and Methods

### 2.1. Study Design and Population

This retrospective longitudinal observational study used routinely collected data from consecutive adults with T2DM assessed in a specialized outpatient clinic between January 2016 and December 2021. For each participant, T0 was the first eligible standardized assessment, T1 was the assessment approximately six months later, and T2 was the assessment approximately twelve months later; T0 was not a single calendar date. The retrospective analysis was approved by the Ethical Committee of the Faculty of Medicine and Pharmacy, University of Oradea (approval code 43, 31 October 2025), and written informed consent for participation and secondary analysis was obtained from all participants, consistent with the declarations.

Of 811 screened records, 809 patients had a standardized baseline assessment and met the general eligibility criteria. Two of these patients lacked baseline UACR, one of the prespecified clustering variables, and were excluded from phenotype derivation. The complete-case clustering cohort therefore comprised 807 participants. Twelve-month data were available for 320 participants.

Clinical phenotypes were derived exclusively from baseline data and were retained unchanged during follow-up. Six-month analyses used all available measurements in the 807-patient cohort, and twelve-month analyses used the 320 participants with T2 data. Because follow-up availability alone cannot exclude selection bias, baseline characteristics were formally compared between participants with and without T2 data.

### 2.2. Eligibility Criteria

Eligible participants were adults aged 18 years or older with a confirmed diagnosis of T2DM who had complete baseline clinical and laboratory assessments together with available six-month follow-up data. Patients with type 1 diabetes mellitus, gestational diabetes mellitus, secondary forms of diabetes, active malignant disease receiving systemic treatment, or acute infectious or inflammatory conditions at baseline were excluded. Individuals with insufficient clinical or laboratory information for the planned analyses were also excluded. For phenotype derivation, patients lacking any of the nine predefined baseline clustering variables were excluded to ensure complete-case clustering and avoid bias related to missing data.

### 2.3. Clinical and Laboratory Variables

Demographic, anthropometric, clinical, biochemical, complication-related, and therapeutic variables routinely collected during outpatient follow-up were extracted from the electronic medical records. Demographic information included age, sex, occupational status, and diabetes duration, while body mass index (BMI) served as the principal anthropometric measure.

The metabolic assessment included fasting plasma glucose, HbA1c, total cholesterol, LDL cholesterol, HDL cholesterol, triglycerides, and the TyG index. TyG was calculated as ln [fasting triglycerides (mg/dL) × fasting glucose (mg/dL)/2], which reproduced the stored values apart from rounding. Renal status was represented by eGFR and UACR, hepatic fibrosis by FIB-4, and inflammation by CRP. FIB-4 was defined as [age (years) × AST (U/L)]/[platelet count (10^9^/L) × √ALT (U/L)]. The shared analysis database contains the calculated eGFR and FIB-4 values but not all raw components or laboratory metadata. A value of 1.10 mg/mmol occurred in 191 of 807 baseline UACR records and was analyzed exactly as stored because the database contained no censoring flag. (1.10 mg/mmol was a lower reporting limit).

Cardiovascular assessment included systolic and diastolic blood pressure together with documentation of hypertension, previous myocardial infarction, and established coronary heart disease. Diabetes-related complications comprised diabetic neuropathy and diabetic retinopathy. Baseline pharmacological therapy was categorized according to the principal glucose-lowering drug classes, including biguanides, sodium-glucose cotransporter-2 (SGLT2) inhibitors, glucagon-like peptide-1 receptor agonists (GLP-1 RAs), dipeptidyl peptidase-4 (DPP-4) inhibitors, sulfonylureas, and insulin therapy. Importantly, cardiovascular variables, diabetes-related complications, and pharmacological treatments were not incorporated into the clustering procedure but were used exclusively for post hoc clinical characterization of the identified phenotypes.

### 2.4. Identification of Data-Derived Clinical Phenotypes

Clinical phenotypes were identified using an unsupervised machine-learning approach based on nine baseline variables selected a priori to represent complementary biological dimensions of T2DM. These variables included diabetes duration, HbA1c, BMI, TyG index, LDL cholesterol, eGFR, UACR, FIB-4 index, and CRP, collectively reflecting disease chronicity, glycemic severity, adiposity, insulin resistance, lipid metabolism, renal function, albuminuria, hepatic fibrosis, and systemic inflammation.

Raw skewness for the nine clustering variables ranged from −0.397 for eGFR to 6.548 for CRP. UACR (skewness 2.219) and CRP (6.548) were transformed using ln(x + 1), reducing skewness to 0.620 and 1.335, respectively; the other seven variables were retained on their original scales and all nine were standardized as sample Z scores. Because diabetes duration, HbA1c, and FIB-4 also showed residual positive skewness, a sensitivity analysis applied ln(x + 1) consistently to all nine positive variables before standardization and four-cluster K-means analysis.

Phenotype identification followed a two-stage exploratory strategy. Ward hierarchical clustering with squared Euclidean distance was examined for two to six clusters. Candidate solutions were additionally quantified using the silhouette coefficient, Calinski–Harabasz index, and Davies–Bouldin index. The final membership used for all manuscript analyses was the 807-patient assignment stored in the database (Cluster_final). Two hundred bootstrap samples were used to refit four-cluster K-means models and compare predicted assignments in the original cohort with the stored solution using the adjusted Rand index.

The agglomeration schedule showed large final fusion increments, but the formal internal indices did not converge on a unique optimum. The four-cluster solution was retained because it produced clinically interpretable profiles of adequate size and matched the prespecified final assignment, while its modest internal separation and stability were treated as limitations rather than evidence of definitive subtype structure.

After clustering, descriptive clinical labels were assigned according to the dominant standardized cluster centroids together with their corresponding untransformed clinical characteristics. Variables such as age, sex, occupational status, cardiovascular disease, diabetes-related complications, and pharmacological treatment were intentionally excluded from phenotype derivation and were evaluated only during subsequent clinical characterization.

### 2.5. Statistical Analysis

Continuous variables were summarized as mean ± standard deviation or median (interquartile range), as appropriate, and categorical variables as number (percentage). The nine variables used to construct the clusters were presented descriptively by phenotype without inferential *p* values because the grouping algorithm was explicitly designed to separate patients on these variables. Omnibus tests were restricted to variables not used for cluster construction and were adjusted across Table 3 using the Benjamini–Hochberg false-discovery-rate procedure.

For non-clustering variables, approximately normal continuous measures were compared using one-way ANOVA, skewed measures using the Kruskal–Wallis test, and categorical measures using Pearson’s chi-square test. Attrition analyses compared baseline characteristics between participants with and without T2 data using Welch’s *t* test, Mann–Whitney U test, or chi-square test, with false-discovery-rate correction.

Standardized cluster centroids were examined to determine the variables contributing most strongly to the clinical identity of each phenotype. Final phenotype interpretation was based on both standardized centroid profiles and the corresponding raw clinical characteristics, thereby ensuring clinical interpretability of the machine-learning-derived clusters.

Raw T1–T0 and T2–T0 changes were retained for descriptive presentation. Formal longitudinal inference used generalized estimating equations with a Gaussian identity link, participant-level clustering, an exchangeable working correlation structure, categorical time, baseline phenotype, and the time-by-phenotype interaction. Triglycerides, UACR, and CRP were analyzed as ln(x + 1). Interaction *p* values across the nine longitudinal outcomes were adjusted using Benjamini–Hochberg false-discovery-rate control. Model-based pairwise contrasts of phenotype-specific change at T1 and T2 were adjusted using Holm’s method within each outcome and time point.

Treatment status at T0 and T2 was summarized within phenotype for insulin, biguanides, sulfonylureas, DPP-4 inhibitors, GLP-1 receptor agonists, and SGLT2 inhibitors. The database did not contain treatment status at T1, medication doses, adherence, or reasons for treatment changes; these limitations were considered when interpreting longitudinal changes. Analyses were reproduced in Python 3.11 (pandas, scikit-learn, scipy, and statsmodels) from the supplied CSV; two-sided adjusted values below 0.05 were considered statistically significant.

## 3. Results

### 3.1. Study Population and Baseline Characteristics of the Phenotype-Derivation Cohort

**Figure 1:**
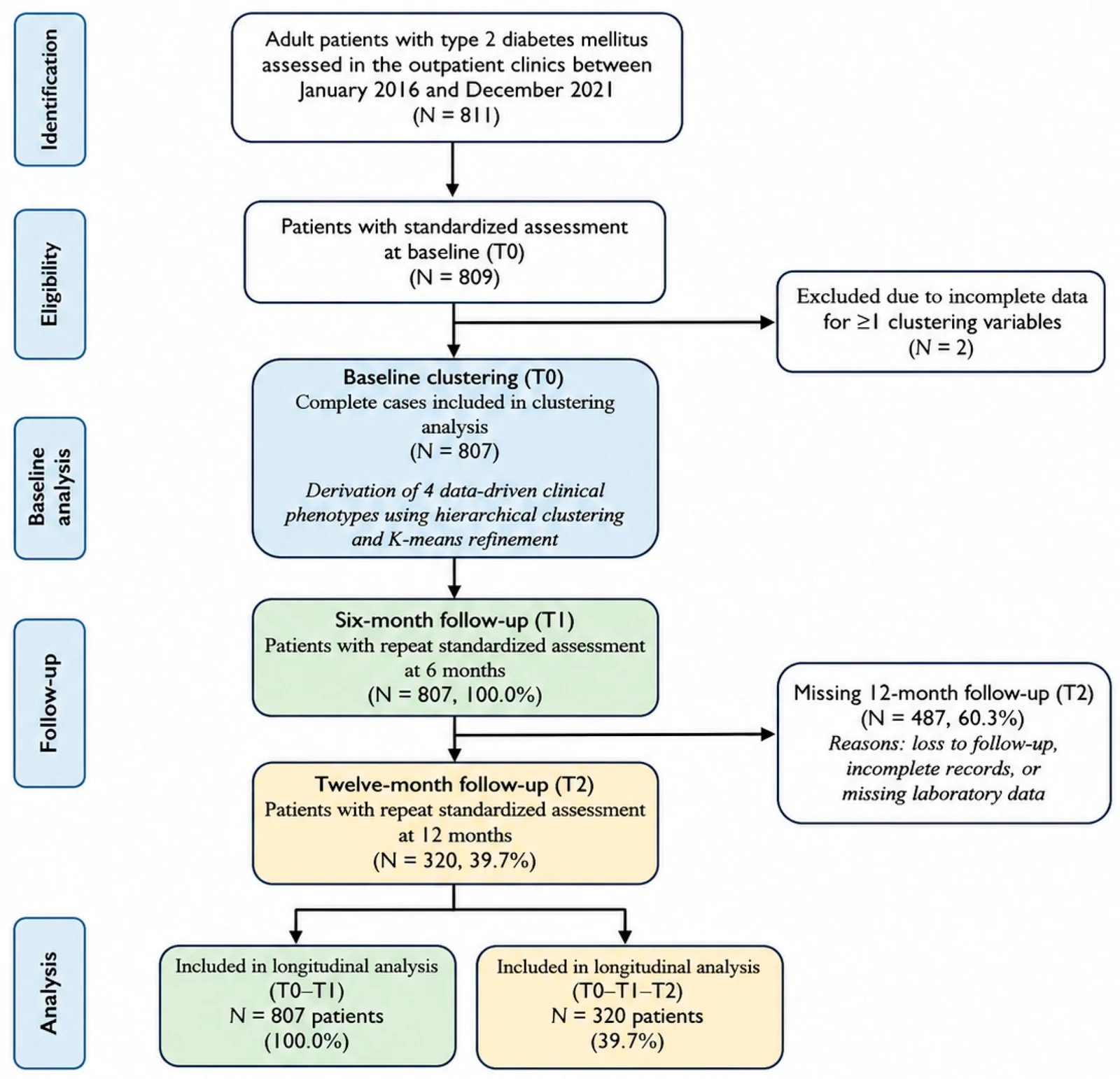
Flow chart of patient selection, phenotype derivation, and longitudinal follow-up. Of 811 screened adults assessed between January 2016 and December 2021, 809 had a standardized baseline assessment; two were excluded because at least one required clustering variable was unavailable, leaving 807 participants for phenotype derivation and six-month analysis. Twelve-month data were available for 320 participants.

Of 811 screened adults, 809 had a standardized baseline assessment and met the general eligibility criteria. Two patients with missing baseline UACR were excluded before clustering, leaving 807 complete cases. This reconciles the screening count in Figure 1 with the 809-patient baseline cohort reported in the Abstract.

Among the included patients, 430 (53.3%) were men and 377 (46.7%) were women. The mean age was 63.32 ± 10.05 years, with values ranging from 33 to 88 years. The median duration of type 2 diabetes was 6 years (IQR 4–10). Most patients were retired (576; 71.4%), whereas 231 (28.6%) were employed.

The baseline metabolic profile showed substantial heterogeneity. Mean HbA1c was 7.95 ± 1.57%, and mean fasting plasma glucose was 176.48 ± 57.50 mg/dL. The mean body mass index was 30.41 ± 5.44 kg/m^2^, indicating a high overall burden of excess adiposity in the cohort.

The lipid profile was characterized by mean total cholesterol of 195.14 ± 56.77 mg/dL, LDL cholesterol of 98.24 ± 33.14 mg/dL, HDL cholesterol of 44.98 ± 12.42 mg/dL, and triglycerides of 164.08 ± 91.53 mg/dL. The mean TyG index was 9.39 ± 0.63, reflecting considerable variability in the degree of insulin resistance.

Renal, inflammatory, and hepatic markers also demonstrated wide interindividual variation. Mean estimated glomerular filtration rate was 79.12 ± 19.50 mL/min/1.73 m^2^, while median UACR was 2.20 mg/mmol (IQR 1.10–8.64). Median C-reactive protein was 1.80 mg/L (IQR 1.00–3.85), and the mean FIB-4 index was 1.28 ± 0.40.

Overall, the complete-case–cohort demonstrated marked heterogeneity across glycemic, anthropometric, lipid, renal, inflammatory, and hepatic domains, supporting the use of multidimensional unsupervised phenotyping. The baseline characteristics of the analytical cohort are presented in Table 1.

### 3.2. Identification and Distribution of the Data-Derived Clinical Phenotypes

Unsupervised clustering of the complete-case analytical cohort identified four mutually exclusive clinical phenotypes among the 807 patients with type 2 diabetes mellitus. Phenotype derivation was based on nine standardized baseline variables representing diabetes duration, glycemic control, adiposity, insulin resistance, lipid status, renal function, albuminuria, hepatic fibrosis, and systemic inflammation.

The final stored four-cluster assignment contained 294, 239, 141, and 133 participants. For this assignment, the silhouette coefficient was 0.118, the Calinski–Harabasz index was 87.6, and the Davies–Bouldin index was 2.118. Across independently refitted K-means solutions, no single candidate from two to six clusters was optimal on all indices. Bootstrap agreement with the stored assignment was moderate (median adjusted Rand index 0.445, IQR 0.407–0.498). The sensitivity analysis using ln(x + 1) for all nine variables showed substantial agreement with the stored four-cluster solution (adjusted Rand index 0.776). These results support use of the four profiles for exploratory description but not as a formally validated classification. The distribution of patients across the four data-derived clinical phenotypes is presented in Table 2.

**Figure 2:**
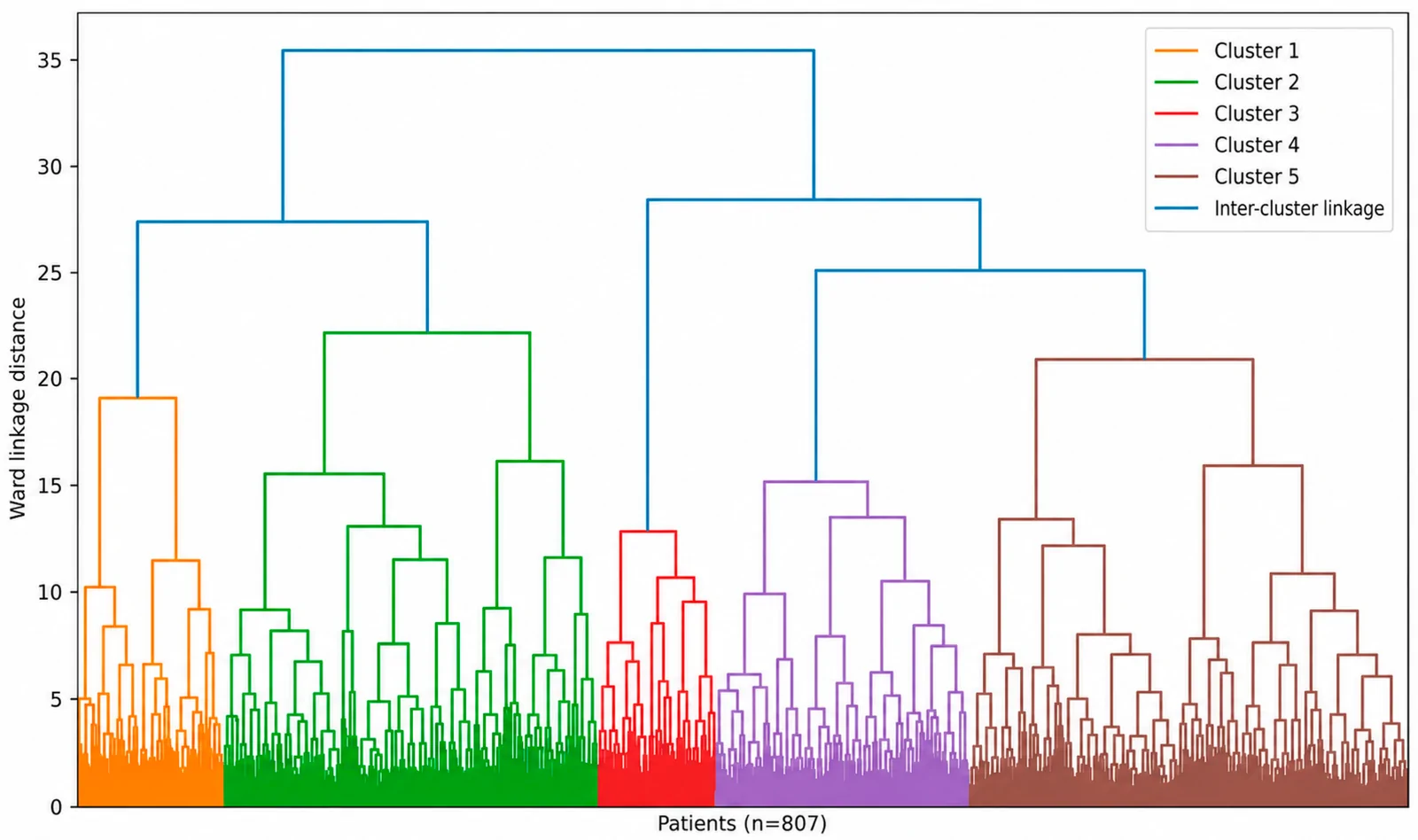
Hierarchical clustering dendrogram supporting the identification of four data-derived clinical phenotypes among patients with type 2 diabetes mellitus. Hierarchical agglomerative clustering was performed in the complete-case–cohort (*n* = 807) using Ward’s minimum variance method. Phenotype derivation was based on nine standardized baseline variables: diabetes duration, HbA1c, body mass index, TyG index, LDL cholesterol, estimated glomerular filtration rate, urinary albumin-to-creatinine ratio, FIB-4 index, and C-reactive protein. UACR and CRP were transformed using ln(x + 1) before Z-score standardization. The dendrogram illustrates the hierarchical structure that supported the subsequent four-cluster solution refined by K-means clustering.

Baseline comparison of the 320 participants with T2 data and the 487 without T2 data found no false-discovery-rate-adjusted differences across 16 demographic, clinical, biochemical, complication, or treatment variables (all q ≥ 0.938; Supplementary Table S2) (Figure 2).

### 3.3. Clinical Characterization of the Data-Derived Phenotypes

The four-cluster solution identified clinically interpretable descriptive profiles (Table 3). The nine cluster-generating variables are reported without *p* values; inferential comparisons and false-discovery-rate-adjusted q values are limited to variables not used to construct the clusters.

The comparatively favorable-profile phenotype comprised 294 patients (36.4%) and had the most favorable combination of measured glycemic, renal, and inflammatory variables within this cohort. This label is descriptive and does not imply demonstrated lower future cardiovascular, renal, or mortality risk.

The cardiorenal–fibrotic phenotype included 239 patients (29.6%) and was characterized by the lowest eGFR, highest UACR, longest diabetes duration, and highest FIB-4. It also had numerically more coronary heart disease and insulin use, but the coronary heart disease difference did not remain significant after false-discovery-rate correction.

The inflammatory–metabolic phenotype comprised 141 patients (17.5%) and was distinguished primarily by pronounced systemic inflammation. Median CRP concentrations were substantially higher than in the remaining phenotypes, while patients also demonstrated higher BMI, triglycerides, TyG index, and LDL cholesterol. Renal impairment was intermediate, suggesting that this phenotype represents a subgroup in which inflammatory activation predominates over overt renal dysfunction.

The severe hyperglycemic–obesity phenotype, consisting of 133 patients (16.5%), exhibited the highest baseline HbA1c, fasting plasma glucose, BMI, and TyG index, consistent with severe glycemic dysregulation and marked insulin resistance. Despite this adverse metabolic profile, renal function remained relatively preserved and albuminuria was generally low compared with the cardiorenal–fibrotic phenotype. This phenotype therefore appeared to be driven predominantly by metabolic rather than renal abnormalities.

**Figure 3:**
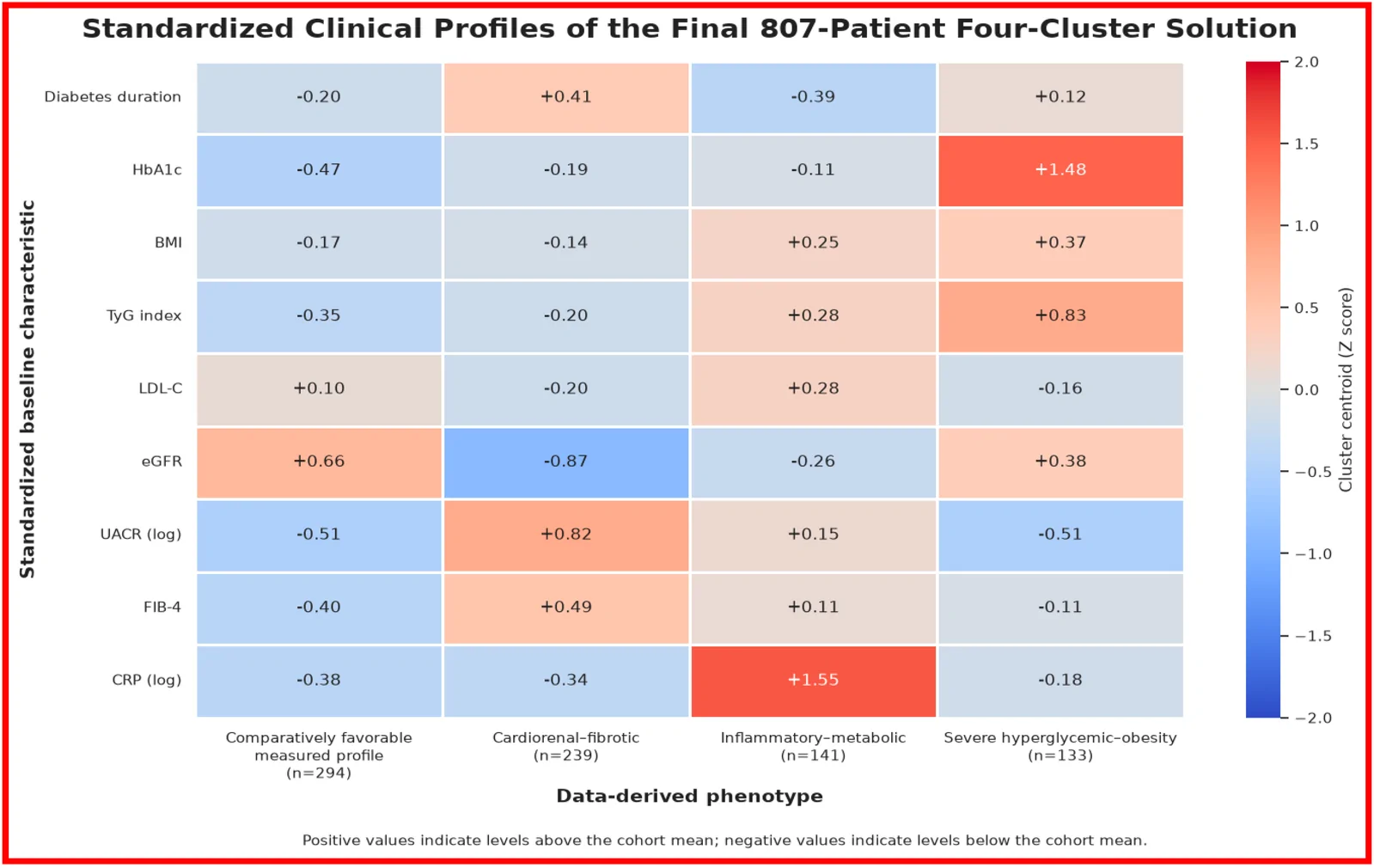
Corrected heatmap of standardized centroids from the final stored 807-patient solution. Cluster sizes are 294, 239, 141, and 133. The corrected plot confirms that the third phenotype is characterized by the highest CRP centroid and supports the inflammatory–metabolic label. Positive values indicate levels above the cohort mean; negative values indicate levels below the cohort mean.

Among variables not used for cluster construction, false-discovery-rate-adjusted differences remained for age, fasting plasma glucose, triglycerides, neuropathy, insulin use, and GLP-1 receptor agonist use (all q < 0.05). DPP-4 inhibitor use (q = 0.054) and coronary heart disease (q = 0.091) did not remain significant after correction. The cluster-generating variables were interpreted descriptively rather than through circular significance testing (Figure 3).

**Figure 4:**
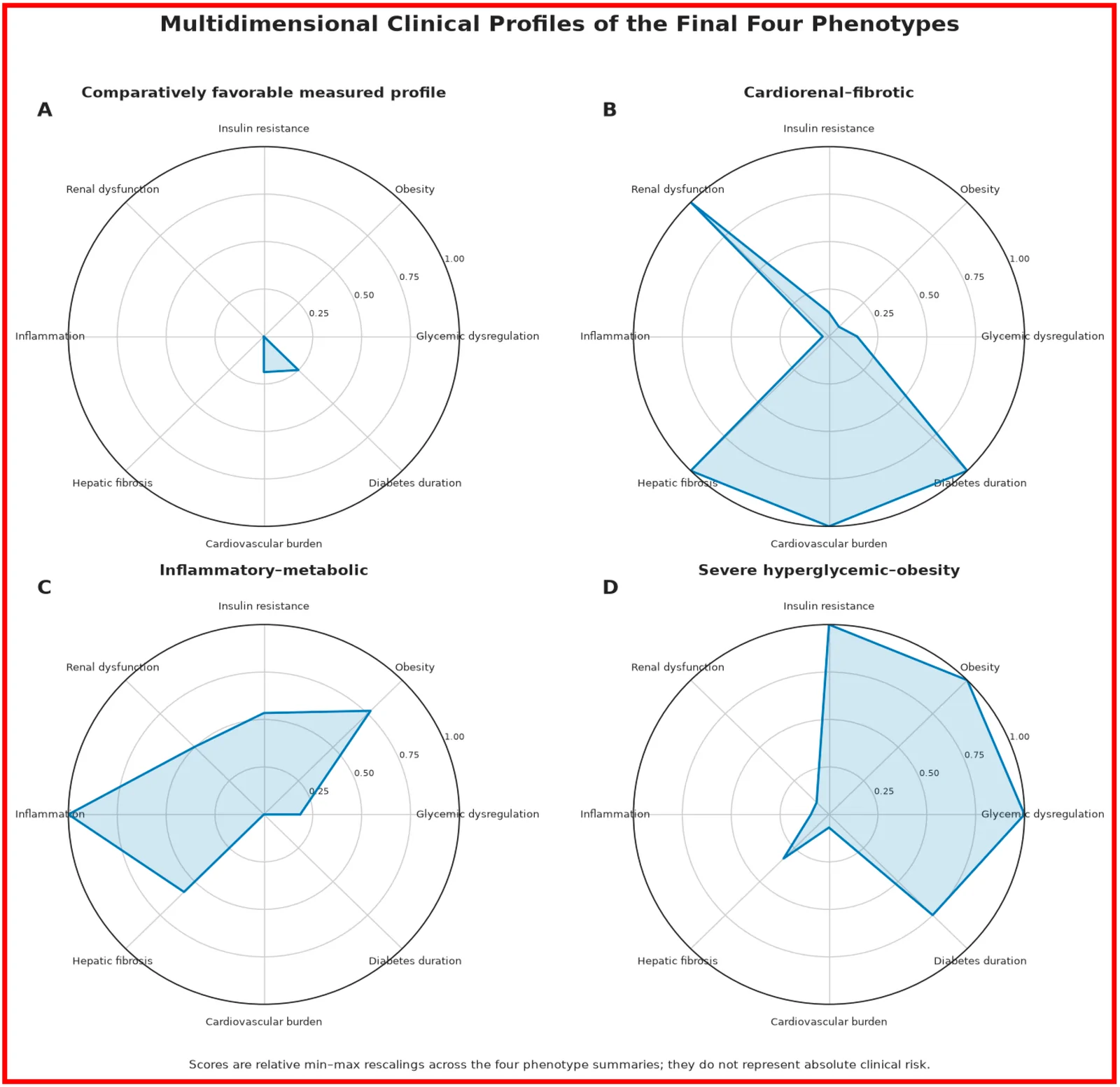
Corrected radar plots of descriptive domains. (**A**) Comparatively favorable measured profile; (**B**) Cardiorenal–fibrotic; (**C**) Inflammatory-metabolic; (**D**) Severe hyperglycemic-obesity. Glycemic dysregulation, obesity, insulin resistance, inflammation, hepatic fibrosis, and diabetes duration use phenotype summaries of HbA1c, BMI, TyG, CRP, FIB-4, and duration, respectively. Renal dysfunction is the mean of rescaled ln(UACR + 1) and inverse eGFR. Cardiovascular burden is the mean phenotype prevalence of hypertension, previous myocardial infarction, and coronary heart disease. Each domain was min–max rescaled from 0 to 1 across only the four phenotype summaries; the plots therefore show relative contrasts and may visually amplify small absolute differences. Cardiovascular burden was used only for visualization and was not included in phenotype derivation.

Figure 4 presents corrected radar plots based on explicitly defined descriptive domains.

### 3.4. Longitudinal Changes According to Baseline Clinical Phenotype

Longitudinal measurements were evaluated using time-by-phenotype generalized estimating equations, while raw change scores were retained for clinical interpretation. Twelve-month analyses included 320 participants. Pairwise model-based contrasts are provided in Supplementary Table S4.

At six months, the severe hyperglycemic–obesity phenotype had the largest raw HbA1c reduction (−0.99 ± 1.24%). The overall time-by-phenotype interaction was significant (q < 0.001), and Holm-adjusted pairwise contrasts showed a greater reduction than in the comparatively favorable-profile, cardiorenal–fibrotic, and inflammatory–metabolic phenotypes. Because this group began with the highest HbA1c and experienced treatment intensification, the magnitude of reduction is compatible with both therapeutic response and regression to the mean.

The inflammatory–metabolic phenotype had the largest raw CRP reduction at T1 and T2. The CRP time-by-phenotype interaction remained significant after correction (q < 0.001), with Holm-adjusted contrasts separating this phenotype from the other three at both time points. This pattern is consistent with change from an unusually high baseline CRP and should not be interpreted as an intrinsic phenotype-specific response.

The cardiorenal–fibrotic phenotype had the largest raw UACR reduction, and the time-by-phenotype interaction for ln(UACR + 1) was significant (q < 0.001). Its eGFR remained the lowest throughout follow-up. These changes may reflect baseline severity, regression to the mean, and increasing use of renally active therapy rather than an effect of phenotype membership itself.

Time-by-phenotype interactions were significant for HbA1c, BMI, TyG, triglycerides, UACR, eGFR, FIB-4, and CRP after false-discovery-rate correction; LDL cholesterol was the only modeled outcome without a significant interaction (q = 0.317).

At twelve months, the severe hyperglycemic–obesity phenotype continued to show the largest raw reductions in HbA1c, BMI, TyG, and triglycerides. Pairwise Holm-adjusted contrasts confirmed larger HbA1c reductions than in each other phenotype and larger BMI reductions than in the other three phenotypes. The inflammatory–metabolic phenotype retained the largest CRP reduction, and the cardiorenal–fibrotic phenotype retained the largest UACR reduction.

**Figure 5:**
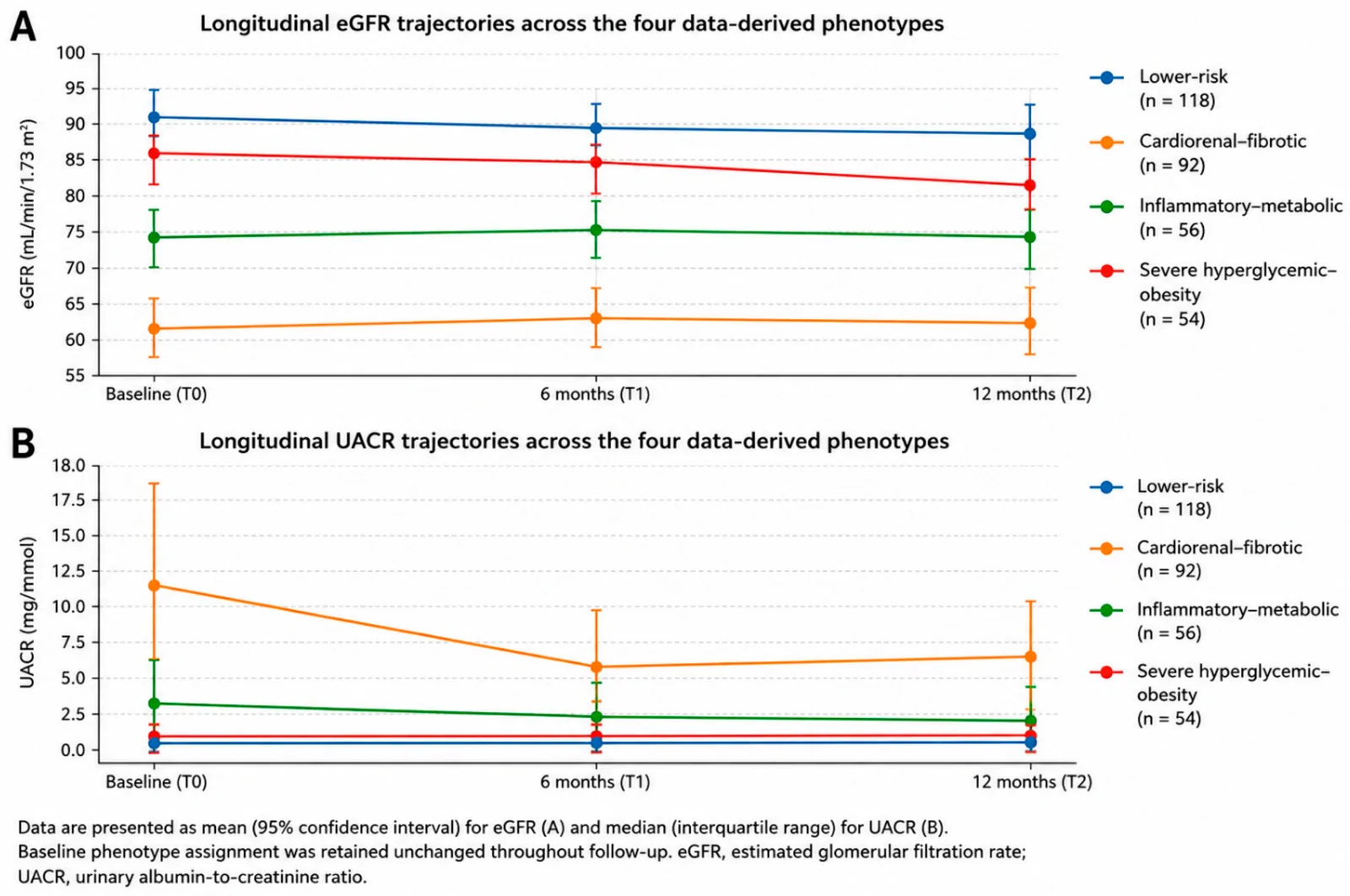
Renal measurements over time according to baseline phenotype. Values are descriptive summaries at T0, T1, and T2. The cardiorenal–fibrotic phenotype retained the lowest eGFR and highest UACR. Between-phenotype differences in change were formally evaluated using time-by-phenotype generalized estimating equations; treatment changes and regression to the mean limit causal interpretation.

Treatment changes were substantial in the 320-patient T2 subgroup. GLP-1 receptor agonist use increased from 53/320 (16.6%) at T0 to 172/320 (53.8%) at T2, SGLT2 inhibitor use from 107/320 (33.4%) to 188/320 (58.8%), biguanide use from 176/320 (55.0%) to 236/320 (73.8%), and insulin use from 85/320 (26.6%) to 98/320 (30.6%). Treatment intensification was especially marked in the severe hyperglycemic–obesity phenotype. T1 medication status and dose changes were unavailable. Accordingly, longitudinal differences cannot be attributed to baseline phenotype alone (Table 4 and Figure 5).

## 4. Discussion

### 4.1. Principal Findings

The present study identified four clinically interpretable, data-derived profiles using nine routinely available baseline variables: a comparatively favorable-profile phenotype, a cardiorenal–fibrotic phenotype, an inflammatory–metabolic phenotype, and a severe hyperglycemic–obesity phenotype. These labels describe the dominant measured features in this cohort and do not define validated biological or prognostic subtypes.

The phenotypes differed substantially in their baseline clinical and biochemical characteristics. The comparatively favorable-profile phenotype demonstrated the most favorable overall profile, with lower HbA1c and TyG index values, preserved renal function, limited albuminuria, and low inflammatory burden. The cardiorenal–fibrotic phenotype combined the lowest eGFR, the highest UACR, longer diabetes duration, and the highest FIB-4 values. The inflammatory–metabolic phenotype was distinguished by markedly elevated CRP concentrations together with an adverse metabolic profile, whereas the severe hyperglycemic–obesity phenotype exhibited the highest HbA1c, fasting plasma glucose, BMI, triglycerides, and TyG index. These patterns indicate that clinically relevant heterogeneity in type 2 diabetes extends beyond glycemic control alone [13,14,15,16].

The phenotype-by-time models identified differential patterns for all longitudinal outcomes except LDL cholesterol. However, the largest improvements generally occurred in the groups with the most abnormal baseline values. This pattern, together with major changes in glucose-lowering therapy, means that regression to the mean and treatment intensification are credible explanations for part of the observed change [17,18,19].

The longitudinal findings should therefore be interpreted as adjusted descriptions of differential change over time, not as evidence that baseline phenotype independently caused improvement or deterioration. The data support phenotype-by-time heterogeneity within this cohort but do not establish phenotype-specific treatment response.

### 4.2. Clinical Significance of the Identified Phenotypes

The comparatively favorable-profile phenotype represented the largest subgroup and had the most favorable combination of the measured glycemic, renal, and inflammatory variables. The name deliberately avoids prognostic language: cardiovascular events, renal endpoints, and mortality were not evaluated, and the profile should not be interpreted as demonstrating lower future risk [20,21,22,23].

The cardiorenal–fibrotic phenotype identified patients with the most pronounced renal involvement, reflected by reduced eGFR and substantially increased albuminuria. The concurrent longer diabetes duration and higher FIB-4 values suggest that renal and hepatic abnormalities may coexist within a subgroup characterized by greater cumulative disease burden. The higher prevalence of coronary heart disease in this phenotype further supports the need for integrated cardiorenal and hepatic assessment rather than glycemic evaluation alone [24].

The inflammatory–metabolic phenotype is an important finding of the present study. Its corrected final centroid profile shows the highest CRP together with higher BMI, triglycerides, and TyG. This profile differs from insulin secretion/insulin resistance subgroups in the Ahlqvist framework because it incorporates a direct inflammatory marker and routine renal and hepatic measures [5,25,26,27].

The severe hyperglycemic–obesity phenotype combined pronounced hyperglycemia, obesity, hypertriglyceridemia, and insulin resistance. Although renal function was relatively preserved at baseline, these patients had the highest prevalence of neuropathy and insulin treatment. The large reduction in HbA1c during follow-up should therefore not be interpreted as indicating a favorable overall prognosis, because it largely reflects regression from markedly elevated baseline values and substantial metabolic burden remained present [28].

Together, these findings demonstrate that patients with type 2 diabetes may share similar diagnostic labels while differing considerably in their dominant clinical vulnerabilities. A multidimensional phenotype-based description may therefore complement conventional evaluation by identifying whether the principal burden is glycemic, renal, inflammatory, hepatic, or mixed.

### 4.3. Comparison with Previous Phenotyping Approaches

Ahlqvist et al. established a foundational five-cluster framework based on autoimmunity, age, BMI, glycemia, beta-cell function, and insulin resistance [5], subsequently replicated in the DD2 cohort and other settings [6]. The present study does not recreate those subtypes because the database lacks GAD antibodies and direct insulin-secretion measures. Instead, it asks whether routinely recorded eGFR, UACR, CRP, and FIB-4 add a pragmatic cardiorenal, inflammatory, and hepatic layer to clinical description.

The inclusion of eGFR and UACR exposed a cardiorenal–fibrotic profile that was not simply the group with the highest glycemic burden, while CRP identified an inflammatory–metabolic profile and FIB-4 contributed a hepatic dimension [29,30]. These additions are clinically accessible but should be viewed as complementary descriptors, not evidence that the new clusters are superior to or interchangeable with established diabetes subgroups.

The study therefore supports a pragmatic multidimensional approach to clinical phenotyping. However, the identified clusters should not be interpreted as definitive biological subtypes. They are data-derived profiles that depend on the selected variables, their preprocessing, the study population, and the clustering method. Replication in independent populations is required before determining whether the same phenotypes are consistently observed in other settings.

### 4.4. Clinical Implications

The identified phenotypes may help clinicians recognize dominant patterns of clinical burden that are not fully captured by HbA1c. Patients belonging to the cardiorenal–fibrotic phenotype may warrant particularly close monitoring of eGFR, UACR, cardiovascular disease, and hepatic fibrosis markers. Those in the inflammatory–metabolic phenotype may require greater attention to obesity, dyslipidemia, insulin resistance, and potential contributors to persistent systemic inflammation [31].

Patients in the severe hyperglycemic–obesity phenotype may benefit from early and intensive metabolic management focused on glycemic control, weight reduction, and insulin resistance. Although this phenotype showed the greatest improvement in HbA1c, its baseline and residual metabolic burden remained substantial. Conversely, the comparatively favorable-profile phenotype may require less intensive short-term escalation but still needs continued monitoring because a comparatively favorable profile does not eliminate long-term diabetes-related risk [1,32].

The present findings do not establish phenotype-specific treatment recommendations and should not replace guideline-based clinical decision-making. Rather, the phenotypes may provide a framework for organizing multidimensional information and prioritizing monitoring according to the dominant abnormalities present in each patient profile.

### 4.5. Strengths and Limitations

This study has several strengths: a relatively large real-world cohort; complete baseline data for nine routinely available variables in 807 participants; simultaneous coverage of glycemic, anthropometric, lipid, renal, inflammatory, and hepatic domains; explicit reconciliation of the 811→809→807 participant flow; formal internal validation across candidate cluster numbers; bootstrap and transformation sensitivity analyses; corrected figures generated from the final stored assignment; phenotype-by-time longitudinal models; adjusted post hoc contrasts; attrition analysis; and direct quantification of available treatment changes.

Several limitations should be considered. The retrospective single-center design limits generalizability and precludes causal inference. Twelve-month data were available for 320/807 participants. Although T2 availability was similar across phenotypes and none of 16 compared baseline characteristics differed after false-discovery-rate correction (all q ≥ 0.938), unmeasured determinants of attrition may still have introduced selection bias.

Cluster validation was modest and did not identify a unique optimal cluster count. The stored four-cluster solution had a silhouette coefficient of 0.118 and median bootstrap-adjusted Rand index of 0.445. Agreement with a consistently transformed four-cluster sensitivity solution was stronger (adjusted Rand index 0.776), but external replication is still required. The original K-means initialization and convergence log were absent from the shared files and must be confirmed from the statistical syntax.

Longitudinal generalized estimating equations addressed the time-by-phenotype question, but they do not remove regression to the mean because the phenotypes were defined using the same baseline variables later followed over time. Treatment also shaped both the baseline clustering variables and subsequent changes. The database documented T0 and T2 drug classes but not T1 therapy, doses, adherence, or non-diabetes medications, so phenotype-specific change cannot be separated from treatment intensification.

Additional limitations include unavailable laboratory methods and eGFR equation in the shared database, an unresolved question about the repeated UACR value of 1.10 mg/mmol, lack of medication adherence, diet, physical activity, socioeconomic data, insulin secretion, direct insulin sensitivity, and hard cardiovascular, renal, or mortality outcomes. The phenotype labels remain post hoc descriptive summaries.

## 5. Conclusions

In this retrospective longitudinal study, nine routinely available baseline variables identified four exploratory profiles: comparatively favorable, cardiorenal–fibrotic, inflammatory–metabolic, and severe hyperglycemic–obesity.

The profiles differed in measured metabolic, renal, inflammatory, hepatic, complication, and treatment characteristics. Time-by-phenotype interactions were observed for most longitudinal measures, but the largest improvements arose from the most abnormal baseline values and coincided with major treatment intensification.

The findings support multidimensional description beyond HbA1c alone, but they do not establish prognostic classes or phenotype-specific treatment effects. Modest internal cluster stability, regression to the mean, treatment changes, attrition, and missing laboratory metadata require cautious interpretation. Independent multicenter validation and prospective outcome studies are required before clinical implementation.

## Figures and Tables

**Table 1:** Baseline demographic, clinical, and biochemical characteristics of the complete-case phenotype-derivation cohort.

Variable	Overall Cohort (*n* = 807)
Age, years	63.32 ± 10.05
Age range, years	33–88
Male sex, *n* (%)	430 (53.3)
Female sex, *n* (%)	377 (46.7)
Diabetes duration, years	6 (4–10)
Employed, *n* (%)	231 (28.6)
Retired, *n* (%)	576 (71.4)
HbA1c, %	7.95 ± 1.57
Fasting plasma glucose, mg/dL	176.48 ± 57.50
Body mass index, kg/m^2^	30.41 ± 5.44
Total cholesterol, mg/dL	195.14 ± 56.77
LDL cholesterol, mg/dL	98.24 ± 33.14
HDL cholesterol, mg/dL	44.98 ± 12.42
Triglycerides, mg/dL	164.08 ± 91.53
TyG index	9.39 ± 0.63
eGFR, mL/min/1.73 m^2^	79.12 ± 19.50
UACR, mg/mmol	2.20 (1.10–8.64)
FIB-4 index	1.28 ± 0.40
C-reactive protein, mg/L	1.80 (1.00–3.85)

Data are presented as mean ± standard deviation, median (interquartile range), range, or number (percentage), as appropriate. Of 811 screened records, 809 had standardized baseline assessments; two of these 809 were excluded from clustering because baseline UACR was unavailable. eGFR, estimated glomerular filtration rate; FIB-4, Fibrosis-4 index; TyG, triglyceride-glucose index; UACR, urinary albumin-to-creatinine ratio.

**Table 2:** Distribution of patients across the four data-derived clinical phenotypes.

Data-Derived Phenotype	Complete-Case Analytical Cohort (*n* = 807), *n* (%)	Patients with 12-Month Follow-Up (*n* = 320), *n* (%)	T2 Availability Within Phenotype (%)
Comparatively favorable-profile phenotype	294 (36.4)	118 (36.9)	40.1
Cardiorenal–fibrotic phenotype	239 (29.6)	92 (28.8)	38.5
Inflammatory–metabolic phenotype	141 (17.5)	56 (17.5)	39.7
Severe hyperglycemic–obesity phenotype	133 (16.5)	54 (16.9)	40.6
Total	807 (100.0)	320 (100.0)	39.7

Percentages in the first two columns were calculated using the corresponding cohort totals. The final column represents the proportion of patients within each baseline phenotype who completed the twelve-month evaluation. Baseline phenotype assignment remained unchanged throughout the longitudinal analyses.

**Table 3:** Baseline clinical, biochemical, complication, and therapeutic characteristics according to the four data-derived phenotypes.

Variable	Comparatively Favorable-Profile Phenotype (*n* = 294)	Cardiorenal–Fibrotic Phenotype (*n* = 239)	Inflammatory–Metabolic Phenotype (*n* = 141)	Severe Hyperglycemic–Obesity Phenotype (*n* = 133)	FDR-Adjusted q Value
Demographic and clinical characteristics					
Age, years	62.38 ± 9.83	66.10 ± 9.76	62.70 ± 10.17	61.03 ± 9.94	<0.001
Male sex, *n* (%)	142 (48.3)	135 (56.5)	85 (60.3)	68 (51.1)	0.148
Diabetes duration, years	6 (3–9)	9 (5–13)	5 (3–8)	8 (4–11)	—
Retired, *n* (%)	205 (69.7)	182 (76.2)	102 (72.3)	87 (65.4)	0.214
Glycemic and anthropometric profile					
HbA1c, %	7.22 ± 0.91	7.66 ± 1.11	7.79 ± 1.12	10.28 ± 1.69	—
Fasting plasma glucose, mg/dL	161.22 ± 46.27	165.23 ± 54.87	183.56 ± 52.54	222.90 ± 64.07	<0.001
BMI, kg/m^2^	29.46 ± 5.02	29.68 ± 4.97	31.75 ± 5.47	32.42 ± 6.28	—
Lipid and insulin-resistance profile					
Total cholesterol, mg/dL	193.09 ± 52.63	192.62 ± 59.31	203.24 ± 56.98	195.60 ± 60.41	0.362
LDL cholesterol, mg/dL	101.49 ± 33.21	91.59 ± 31.05	107.59 ± 34.84	93.11 ± 31.78	—
HDL cholesterol, mg/dL	45.97 ± 12.11	44.95 ± 12.67	44.29 ± 13.17	43.53 ± 11.79	0.345
Triglycerides, mg/dL	139.22 ± 64.30	156.72 ± 94.14	179.57 ± 88.39	215.86 ± 115.22	<0.001
TyG index	9.17 ± 0.56	9.27 ± 0.63	9.57 ± 0.51	9.92 ± 0.56	—
Renal, inflammatory, and hepatic profile					
eGFR, mL/min/1.73 m^2^	91.94 ± 12.92	62.19 ± 14.92	74.01 ± 17.00	86.62 ± 16.56	—
UACR, mg/mmol	1.10 (1.10–2.20)	11.47 (4.43–17.50)	3.30 (1.10–11.20)	1.10 (1.00–2.60)	—
FIB-4 index	1.11 ± 0.29	1.47 ± 0.44	1.32 ± 0.44	1.23 ± 0.36	—
CRP, mg/L	1.20 (1.00–2.30)	1.30 (1.00–2.80)	7.80 (5.80–10.70)	1.50 (1.00–3.10)	—
Cardiovascular and microvascular complications					
Hypertension, *n* (%)	163 (55.4)	141 (59.0)	73 (51.8)	72 (54.1)	0.627
Previous myocardial infarction, *n* (%)	9 (3.1)	10 (4.2)	3 (2.1)	6 (4.5)	0.674
Coronary heart disease, *n* (%)	32 (10.9)	43 (18.0)	18 (12.8)	12 (9.0)	0.091
Retinopathy, *n* (%)	60 (20.4)	61 (25.5)	22 (15.6)	28 (21.1)	0.214
Neuropathy, *n* (%)	76 (25.9)	58 (24.3)	37 (26.2)	54 (40.6)	0.014
Baseline antidiabetic treatment					
Insulin, *n* (%)	57 (19.4)	69 (28.9)	32 (22.7)	49 (36.8)	0.004
Biguanides, *n* (%)	171 (58.2)	147 (61.5)	80 (56.7)	75 (56.4)	0.723
Sulfonylureas, *n* (%)	23 (7.8)	30 (12.6)	9 (6.4)	9 (6.8)	0.180
DPP-4 inhibitors, *n* (%)	53 (18.0)	52 (21.8)	20 (14.2)	13 (9.8)	0.054
GLP-1 receptor agonists, *n* (%)	52 (17.7)	26 (10.9)	35 (24.8)	19 (14.3)	0.014
SGLT2 inhibitors, *n* (%)	92 (31.3)	85 (35.6)	50 (35.5)	54 (40.6)	0.362

Data are presented as mean ± standard deviation, median (interquartile range), or number (percentage). The nine cluster-generating variables (diabetes duration, HbA1c, BMI, TyG, LDL cholesterol, eGFR, UACR, FIB-4, and CRP) are descriptive only and are shown without inferential tests. For variables not used in clustering, the final column reports Benjamini–Hochberg false-discovery-rate-adjusted q values. BMI, body mass index; CRP, C-reactive protein; DPP-4, dipeptidyl peptidase-4; eGFR, estimated glomerular filtration rate; GLP-1, glucagon-like peptide-1; SGLT2, sodium-glucose cotransporter-2; TyG, triglyceride-glucose index; UACR, urinary albumin-to-creatinine ratio.

**Table 4:** Changes in clinical and biochemical parameters according to baseline data-derived phenotype.

**Panel A. Changes from Baseline to Six Months**					
**Parameter, ΔT1–T0**	**Comparatively Favorable-Profile Phenotype (*n* = 294)**	**Cardiorenal–Fibrotic Phenotype (*n* = 239)**	**Inflammatory–Metabolic Phenotype (*n* = 141)**	**Severe Hyperglycemic–Obesity Phenotype (*n* = 133)**	**Interaction q**
HbA1c, %	−0.37 ± 0.76 ^1^	−0.57 ± 0.86	−0.65 ± 0.73	−0.99 ± 1.24	<0.001
BMI, kg/m ^2^	−0.69 ± 1.18	−0.71 ± 1.32	−0.74 ± 1.33	−0.84 ± 1.39	0.007
TyG index	−0.05 ± 0.59	−0.00 ± 0.64	−0.24 ± 0.53	−0.29 ± 0.55	<0.001
Triglycerides, mg/dL	−4.11 ± 62.29	−6.46 ± 63.79	−11.77 ± 65.75	−25.35 ± 74.82	0.002
LDL cholesterol, mg/dL	−11.61 ± 40.17	−5.57 ± 36.35	−14.75 ± 51.04	−8.56 ± 38.95	0.317
UACR, mg/mmol ^2^	0.00 (−1.10 to 1.00)	−5.08 (−12.70 to 0.32)	−0.76 (−5.50 to 0.60)	0.00 (−0.80 to 1.10)	<0.001
eGFR, mL/min/1.73 m ^2^	−2.87 ± 9.30	+1.04 ± 10.71	+2.26 ± 11.23	−0.63 ± 8.87	<0.001
FIB-4 index	+0.071 ± 0.074	+0.069 ± 0.080	+0.050 ± 0.088	+0.050 ± 0.080	0.002
CRP, mg/L ^2^	0.00 (−0.30 to 1.30) ^1^	0.00 (−0.41 to 0.85)	−4.10 (−7.30 to −1.03)	0.00 (−0.50 to 0.90)	<0.001
**Panel B. Changes from baseline to twelve months in the longitudinal subgroup**					
**Parameter, ΔT2–T0**	**Comparatively favorable-profile phenotype (*n* = 118)**	**Cardiorenal–fibrotic phenotype (*n* = 92)**	**Inflammatory–metabolic phenotype (*n* = 56)**	**Severe hyperglycemic–obesity phenotype (*n* = 54)**	**Interaction q**
HbA1c, %	−0.41 ± 0.83	−0.88 ± 0.94	−0.97 ± 0.74	−1.76 ± 0.99	<0.001
BMI, kg/m ^2^	−0.97 ± 1.34	−1.06 ± 1.60	−1.21 ± 1.41	−1.74 ± 1.55	0.007
TyG index	−0.08 ± 0.66	−0.06 ± 0.69	−0.37 ± 0.61	−0.61 ± 0.75	<0.001
Triglycerides, mg/dL ^2^	−15.00 (−45.50 to 30.50)	−15.50 (−47.50 to 15.25)	−36.00 (−69.50 to 13.25)	−53.50 (−96.25 to 10.50)	0.002
LDL cholesterol, mg/dL	−4.72 ± 38.57	−1.30 ± 32.46	−8.55 ± 42.41	−8.61 ± 35.63	0.317
UACR, mg/mmol ^2^	+0.20 (−0.98 to 1.17)	−4.46 (−11.77 to 1.93)	−1.08 (−6.50 to 0.10)	+0.10 (−1.40 to 1.30)	<0.001
eGFR, mL/min/1.73 m ^2^	−3.08 ± 9.02	−0.51 ± 11.39	+1.06 ± 10.63	−4.64 ± 10.38	<0.001
FIB-4 index	+0.039 ± 0.105	+0.045 ± 0.107	−0.002 ± 0.092	−0.013 ± 0.097	0.002
CRP, mg/L ^2^	0.00 (−0.60 to 0.85)	+0.05 (−0.60 to 0.90)	−4.10 (−7.33 to −1.08)	+0.20 (−0.50 to 1.28)	<0.001

Data are presented as mean ± standard deviation or median (interquartile range). Raw changes are descriptive. The final column reports the false-discovery-rate-adjusted q value for the overall time-by-phenotype interaction from a generalized estimating equation fitted across T0, T1, and T2. Triglycerides, UACR, and CRP were modeled after ln(x + 1) transformation. Pairwise Holm-adjusted contrasts are reported in Supplementary Table S4. ^1^ One six-month value was unavailable in the comparatively favorable-profile phenotype; HbA1c and CRP analyses therefore included 293 patients in this group. ^2^ Raw values are summarized as median (interquartile range); formal longitudinal inference used ln(x + 1)-transformed values in the generalized estimating equations.

## Data Availability

The original contributions presented in this study are included in this article. Further inquiries can be directed to the first authors.

## Supplementary Materials

The following supporting information can be downloaded at: https://www.mdpi.com/article/10.3390/biomedicines14091903/s1, Table S1A: Internal validation indices for independently refitted K-means candidate solutions; Table S1B: Distributional assessment and consistent-transformation sensitivity analysis; Table S2: Baseline characteristics of participants with and without twelve-month follow-up; Table S3: Glucose-lowering treatment changes from baseline to twelve months by phenotype; Table S4: Model-based pairwise contrasts of phenotype-specific change; Holm-adjusted *p* values; Table S5: Definitions of radar-plot domains.

## Abbreviations

The following abbreviations are used in this manuscript:

BMI	Body mass index
CI	Confidence interval
CRP	C-reactive protein
DPP-4	Dipeptidyl peptidase-4
eGFR	Estimated glomerular filtration rate
FIB-4	Fibrosis-4 index
GLP-1	Glucagon-like peptide-1
HbA1c	Glycated hemoglobin
HDL	High-density lipoprotein
IQR	Interquartile range
LDL	Low-density lipoprotein
SD	Standard deviation
SGLT2	Sodium–glucose cotransporter-2
T0	Baseline assessment
T1	Six-month follow-up assessment
T2DM	Type 2 diabetes mellitus
TyG	Triglyceride–glucose index
UACR	Urinary albumin-to-creatinine ratio
ARI	Adjusted Rand index
FDR	False discovery rate
GEE	Generalized estimating equation
