# Supplementary material for: Data-Driven Multidimensional Clinical Phenotypes and Longitudinal Changes in Type 2 Diabetes Mellitus: A Retrospective Cohort Study
Source: Biomedicines. 2026 Aug 26;14(9):1903. doi: 10.3390/biomedicines14091903 (PMC13604856; doi:10.3390/biomedicines14091903)
Supplement: Supplementary file 1 [file biomedicines-14-01903-s001.zip › biomedicines-4518012-supplementary.pdf]

Supplementary Files

Table S1A. Internal validation indices for independently refitted K-means candidate solutions.

Clusters	Cluster sizes	Silhouette	Calinski–Harabasz	Davies–Bouldin
2	345/462	0.132	115.3	2.521
3	169/341/297	0.112	95.8	2.294
4	150/332/232/93	0.126	89.5	2.127
5	105/134/242/128/198	0.118	84.7	1.993
6	100/181/226/119/79/102	0.123	79.6	1.891

Table S1B. Distributional assessment and consistent-transformation sensitivity analysis.

Variable/analysis	Raw skewness	Analysis scale	Result
Diabetes duration	1.013	untransformed	1.013
HbA1c	1.326	untransformed	1.326
BMI	0.722	untransformed	0.722
TyG index	-0.070	untransformed	-0.070
LDL cholesterol	0.758	untransformed	0.758
eGFR	-0.397	untransformed	-0.397
UACR	2.219	ln(x+1)	0.620
FIB-4	1.361	untransformed	1.361
CRP	6.548	ln(x+1)	1.335
Sensitivity: ln(x+1) applied to all nine variables	—	Four-cluster K-means	ARI vs stored=0.776; silhouette=0.108

Table S2. Baseline characteristics of participants with and without twelve-month follow-up.

Variable	T2 available (n=320)	T2 unavailable (n=487)	p value	FDR q value
Age, years	63.08 ± 10.17	63.47 ± 9.97	0.586	0.938
Diabetes duration, years	7.00 (4.00–10.00)	6.00 (4.00–10.00)	0.234	0.938
HbA1c, %	7.96 ± 1.61	7.95 ± 1.55	0.872	0.938
BMI, kg/m²	30.55 ± 5.71	30.32 ± 5.26	0.579	0.938
TyG index	9.35 ± 0.64	9.42 ± 0.63	0.165	0.938
LDL cholesterol, mg/dL	98.76 ± 31.82	97.90 ± 34.02	0.715	0.938
eGFR, mL/min/1.73 m²	79.62 ± 18.97	78.79 ± 19.85	0.550	0.938
UACR, mg/mmol	2.20 (1.10–7.95)	2.20 (1.10–9.28)	0.488	0.938
FIB-4 index	1.27 ± 0.39	1.28 ± 0.41	0.885	0.938
CRP, mg/L	1.90 (1.00–3.90)	1.60 (1.00–3.80)	0.292	0.938
Male sex, n (%)	169 (52.8)	261 (53.6)	0.828	0.938
Insulin, n (%)	85 (26.6)	122 (25.1)	0.631	0.938
SGLT2 inhibitor, n (%)	107 (33.4)	174 (35.7)	0.504	0.938

GLP-1 receptor agonist, n (%)	53 (16.6)	79 (16.2)	0.898	0.938
Coronary heart disease, n (%)	42 (13.1)	63 (12.9)	0.938	0.938
Neuropathy, n (%)	92 (28.7)	133 (27.3)	0.655	0.938

Table S3. Glucose-lowering treatment changes from baseline to twelve months by phenotype.

Cluster	Phenotype	Therapy	T0, n (%)	T2, n (%)	Initiated	Discontinued
1	Comparatively favorable measured profile	Insulin	20 (16.9)	20 (16.9)	2	2
1	Comparatively favorable measured profile	Biguanides	64 (54.2)	77 (65.3)	14	1
1	Comparatively favorable measured profile	Sulfonylureas	13 (11.0)	11 (9.3)	0	2
1	Comparatively favorable measured profile	DPP-4 inhibitors	17 (14.4)	17 (14.4)	0	0
1	Comparatively favorable measured profile	GLP-1 receptor agonists	24 (20.3)	53 (44.9)	29	0
1	Comparatively favorable measured profile	SGLT2 inhibitors	40 (33.9)	59 (50.0)	19	0
2	Cardiorenal–fibrotic	Insulin	26 (28.3)	29 (31.5)	4	1
2	Cardiorenal–fibrotic	Biguanides	58 (63.0)	73 (79.3)	16	1
2	Cardiorenal–fibrotic	Sulfonylureas	12 (13.0)	11 (12.0)	0	1
2	Cardiorenal–fibrotic	DPP-4 inhibitors	27 (29.3)	27 (29.3)	0	0
2	Cardiorenal–fibrotic	GLP-1 receptor agonists	8 (8.7)	43 (46.7)	35	0
2	Cardiorenal–fibrotic	SGLT2 inhibitors	28 (30.4)	56 (60.9)	28	0
3	Inflammatory–metabolic	Insulin	18 (32.1)	19 (33.9)	2	1
3	Inflammatory–metabolic	Biguanides	27 (48.2)	39 (69.6)	13	1
3	Inflammatory–metabolic	Sulfonylureas	0 (0.0)	0 (0.0)	0	0
3	Inflammatory–metabolic	DPP-4 inhibitors	9 (16.1)	9 (16.1)	0	0
3	Inflammatory–metabolic	GLP-1 receptor agonists	14 (25.0)	31 (55.4)	17	0
3	Inflammatory–metabolic	SGLT2 inhibitors	21 (37.5)	31 (55.4)	10	0
4	Severe hyperglycemic–obesity	Insulin	21 (38.9)	30 (55.6)	9	0

4	Severe hyperglycemic–obesity	Biguanides	27 (50.0)	47 (87.0)	20	0
4	Severe hyperglycemic–obesity	Sulfonylureas	4 (7.4)	4 (7.4)	0	0
4	Severe hyperglycemic–obesity	DPP-4 inhibitors	4 (7.4)	4 (7.4)	0	0
4	Severe hyperglycemic–obesity	GLP-1 receptor agonists	7 (13.0)	45 (83.3)	38	0
4	Severe hyperglycemic–obesity	SGLT2 inhibitors	18 (33.3)	42 (77.8)	24	0

Table S4. Model-based pairwise contrasts of phenotype-specific change; Holm-adjusted p values.

Outcome	Time	Phenotype comparison	Difference in change	Model scale	Holm p
HbA1c	T1	1 vs 2	+0.200	original	0.014
HbA1c	T1	1 vs 3	+0.281	original	<0.001
HbA1c	T1	1 vs 4	+0.620	original	<0.001
HbA1c	T1	2 vs 3	+0.081	original	0.323
HbA1c	T1	2 vs 4	+0.421	original	0.002
HbA1c	T1	3 vs 4	+0.339	original	0.014
HbA1c	T2	1 vs 2	+0.406	original	<0.001
HbA1c	T2	1 vs 3	+0.502	original	<0.001
HbA1c	T2	1 vs 4	+1.286	original	<0.001
HbA1c	T2	2 vs 3	+0.096	original	0.376
HbA1c	T2	2 vs 4	+0.880	original	<0.001
HbA1c	T2	3 vs 4	+0.784	original	<0.001
BMI	T1	1 vs 2	+0.021	original	1.000
BMI	T1	1 vs 3	+0.054	original	1.000
BMI	T1	1 vs 4	+0.151	original	1.000
BMI	T1	2 vs 3	+0.033	original	1.000
BMI	T1	2 vs 4	+0.130	original	1.000
BMI	T1	3 vs 4	+0.097	original	1.000
BMI	T2	1 vs 2	+0.191	original	0.791
BMI	T2	1 vs 3	+0.196	original	0.791
BMI	T2	1 vs 4	+0.779	original	<0.001
BMI	T2	2 vs 3	+0.005	original	0.979
BMI	T2	2 vs 4	+0.588	original	0.045
BMI	T2	3 vs 4	+0.583	original	0.047
TyG	T1	1 vs 2	-0.043	original	0.850
TyG	T1	1 vs 3	+0.190	original	0.002
TyG	T1	1 vs 4	+0.241	original	<0.001
TyG	T1	2 vs 3	+0.233	original	<0.001
TyG	T1	2 vs 4	+0.284	original	<0.001
TyG	T1	3 vs 4	+0.051	original	0.850
TyG	T2	1 vs 2	-0.009	original	0.895
TyG	T2	1 vs 3	+0.269	original	0.004

TyG	T2	1 vs 4	+0.529	original	<0.001
TyG	T2	2 vs 3	+0.279	original	0.004
TyG	T2	2 vs 4	+0.538	original	<0.001
TyG	T2	3 vs 4	+0.260	original	0.035
Triglycerides	T1	1 vs 2	+0.006	ln(x+1)	0.884
Triglycerides	T1	1 vs 3	+0.091	ln(x+1)	0.190
Triglycerides	T1	1 vs 4	+0.142	ln(x+1)	0.017
Triglycerides	T1	2 vs 3	+0.086	ln(x+1)	0.215
Triglycerides	T1	2 vs 4	+0.136	ln(x+1)	0.027
Triglycerides	T1	3 vs 4	+0.050	ln(x+1)	0.691
Triglycerides	T2	1 vs 2	+0.038	ln(x+1)	0.491
Triglycerides	T2	1 vs 3	+0.153	ln(x+1)	0.098
Triglycerides	T2	1 vs 4	+0.334	ln(x+1)	<0.001
Triglycerides	T2	2 vs 3	+0.115	ln(x+1)	0.216
Triglycerides	T2	2 vs 4	+0.296	ln(x+1)	0.002
Triglycerides	T2	3 vs 4	+0.181	ln(x+1)	0.142
LDL cholesterol	T1	1 vs 2	-6.043	original	0.360
LDL cholesterol	T1	1 vs 3	+3.140	original	1.000
LDL cholesterol	T1	1 vs 4	-3.056	original	1.000
LDL cholesterol	T1	2 vs 3	+9.183	original	0.360
LDL cholesterol	T1	2 vs 4	+2.987	original	1.000
LDL cholesterol	T1	3 vs 4	-6.195	original	1.000
LDL cholesterol	T2	1 vs 2	-7.787	original	0.368
LDL cholesterol	T2	1 vs 3	+2.628	original	1.000
LDL cholesterol	T2	1 vs 4	-1.505	original	1.000
LDL cholesterol	T2	2 vs 3	+10.415	original	0.354
LDL cholesterol	T2	2 vs 4	+6.281	original	0.828
LDL cholesterol	T2	3 vs 4	-4.133	original	1.000
UACR	T1	1 vs 2	+0.792	ln(x+1)	<0.001
UACR	T1	1 vs 3	+0.540	ln(x+1)	<0.001
UACR	T1	1 vs 4	-0.021	ln(x+1)	0.821
UACR	T1	2 vs 3	-0.251	ln(x+1)	0.100
UACR	T1	2 vs 4	-0.812	ln(x+1)	<0.001
UACR	T1	3 vs 4	-0.561	ln(x+1)	<0.001
UACR	T2	1 vs 2	+0.812	ln(x+1)	<0.001
UACR	T2	1 vs 3	+0.664	ln(x+1)	<0.001
UACR	T2	1 vs 4	+0.002	ln(x+1)	0.985
UACR	T2	2 vs 3	-0.148	ln(x+1)	0.675
UACR	T2	2 vs 4	-0.810	ln(x+1)	<0.001
UACR	T2	3 vs 4	-0.662	ln(x+1)	<0.001
eGFR	T1	1 vs 2	-3.911	original	<0.001
eGFR	T1	1 vs 3	-5.129	original	<0.001
eGFR	T1	1 vs 4	-2.238	original	0.068
eGFR	T1	2 vs 3	-1.218	original	0.297
eGFR	T1	2 vs 4	+1.673	original	0.210
eGFR	T1	3 vs 4	+2.891	original	0.068

eGFR	T2	1 vs 2	-3.770	original	0.006
eGFR	T2	1 vs 3	-5.232	original	0.001
eGFR	T2	1 vs 4	+0.384	original	0.778
eGFR	T2	2 vs 3	-1.462	original	0.704
eGFR	T2	2 vs 4	+4.154	original	0.021
eGFR	T2	3 vs 4	+5.616	original	0.005
FIB-4	T1	1 vs 2	+0.001	original	1.000
FIB-4	T1	1 vs 3	+0.021	original	0.076
FIB-4	T1	1 vs 4	+0.020	original	0.076
FIB-4	T1	2 vs 3	+0.020	original	0.109
FIB-4	T1	2 vs 4	+0.019	original	0.109
FIB-4	T1	3 vs 4	-0.001	original	1.000
FIB-4	T2	1 vs 2	-0.004	original	0.747
FIB-4	T2	1 vs 3	+0.033	original	0.036
FIB-4	T2	1 vs 4	+0.050	original	0.002
FIB-4	T2	2 vs 3	+0.037	original	0.032
FIB-4	T2	2 vs 4	+0.054	original	0.002
FIB-4	T2	3 vs 4	+0.016	original	0.595
CRP	T1	1 vs 2	+0.086	ln(x+1)	0.330
CRP	T1	1 vs 3	+0.947	ln(x+1)	<0.001
CRP	T1	1 vs 4	+0.072	ln(x+1)	0.535
CRP	T1	2 vs 3	+0.860	ln(x+1)	<0.001
CRP	T1	2 vs 4	-0.014	ln(x+1)	0.827
CRP	T1	3 vs 4	-0.874	ln(x+1)	<0.001
CRP	T2	1 vs 2	+0.014	ln(x+1)	1.000
CRP	T2	1 vs 3	+0.874	ln(x+1)	<0.001
CRP	T2	1 vs 4	-0.017	ln(x+1)	1.000
CRP	T2	2 vs 3	+0.860	ln(x+1)	<0.001
CRP	T2	2 vs 4	-0.031	ln(x+1)	1.000
CRP	T2	3 vs 4	-0.892	ln(x+1)	<0.001

Table S5. Definitions of radar-plot domains.

Domain	Construction	Transformation/interpretation
Glycemic dysregulation	Mean HbA1c by phenotype	Min–max across four phenotype means
Obesity	Mean BMI by phenotype	Min–max across four phenotype means
Insulin resistance	Mean TyG by phenotype	Min–max across four phenotype means
Renal dysfunction	Average of min–max ln(UACR+1) and min–max inverse eGFR	Equal weighting of the two renal components
Inflammation	Median CRP by phenotype	ln(x+1), then min–max
Hepatic fibrosis	Mean FIB-4 by phenotype	Min–max across four phenotype means
Cardiovascular burden	Mean prevalence of hypertension, previous MI, and coronary heart disease	Descriptive only; not a clustering variable

Diabetes duration	Median duration by phenotype	Min–max across four phenotype medians

Phenotype numbers in Supplementary Tables S3 and S4 correspond to: 1, comparatively favorable-profile; 2, cardiorenal–fibrotic; 3, inflammatory–metabolic; and 4, severe hyperglycemic–obesity. All supplementary analyses were generated directly from the supplied 809-row CSV, with clustering analyses restricted to the 807 complete cases.
